# A 110 Year Sediment Record of Polycyclic Aromatic Hydrocarbons Related to Economic Development and Energy Consumption in Dongping Lake, North China

**DOI:** 10.3390/molecules26226828

**Published:** 2021-11-11

**Authors:** Wei Guo, Junhui Yue, Qian Zhao, Jun Li, Xiangyi Yu, Yan Mao

**Affiliations:** 1 Key Laboratory of Beijing for Water Quality Science and Water Environment Recovery Engineering, Faculty of Architecture, Civil and Transportation Engineering, Beijing University of Technology, Beijing 100124, China; yuejunhui3214@163.com (J.Y.); ZQ1176170762@163.com (Q.Z.); jglijun@bjut.edu.cn (J.L.); 2 Solid Waste and Chemicals Management Center of MEE, Beijing, 100029, China; yuxiangyi@meescc.cn

**Keywords:** PAHs, historical trends, shallow lake, economic parameters, sources, sediment core

## Abstract

A sedimentary record of the 16 polycyclic aromatic hydrocarbon (PAH) pollutants from Dongping Lake, north China, is presented in this study. The influence of regional energy structure changes for 2–6-ring PAHs was investigated, in order to assess their sources and the impact of socioeconomic developments on the observed changes in concentration over time. The concentration of the ΣPAH_16_ ranged from 77.6 to 628.0 ng/g. Prior to the 1970s, the relatively low concentration of ΣPAH_16_ and the average presence of 44.4% 2,3-ring PAHs indicated that pyrogenic combustion from grass, wood, and coal was the main source of PAHs. The rapid increase in the concentration of 2,3-ring PAHs between the 1970s and 2006 was attributed to the growth of the urban population and the coal consumption, following the implementation of the Reform and Open Policy in 1978. The source apportionment, which was assessed using a positive matrix factorization model, revealed that coal combustion was the most important regional source of PAHs pollution (>51.0%). The PAHs were mainly transported to the site from the surrounding regions by atmospheric deposition rather than direct discharge.

## 1. Introduction

Polycyclic aromatic hydrocarbons (PAHs) are a class of persistent organic pollutants (POPs) which are ubiquitously present in the environment [1]. Because of their potential as carcinogens, mutagens, and teratogens [2], PAHs have elicited serious concern worldwide [3,4]. Sixteen PAH compounds have been included in the priority pollutant list for risk control and management of the US Environmental Protection Agency (US EPA) [5]. PAHs originate mainly from the incomplete combustion and/or pyro-synthesis of organic materials through fossil-fuel usage, biomass burning, industrial processes, waste incineration, and vehicle exhaust emissions [6,7,8]. PAHs can be transported into aquatic ecosystems through various processes, including atmospheric deposition [9], wastewater discharge from urban sewage treatment plants, as well as industrial sites [10], or surface runoff from urban or industrial areas [11]. The PAHs discharged into the aquatic environment are prone to combining with fine particles, being ultimately deposited into sediments due to their hydrophobic nature and resulting low solubility [12,13]. This makes sediments an important sink for PAHs, which directly affects the dwelling organisms and aquatic environment safety [14,15,16]. 

PAHs in dated sediments provided an ideal archive of historical information about the anthropogenic contamination of aquatic ecosystems and emission of pollutants from energy consumption [17,18,19,20,21]. Thus, a dated sediment core analysis can reconstruct very well the chronology of PAHs pollution and clearly identify their fate in any water-related ecosystem [22,23,24]. China is currently the world's second largest economy and has undergone a rapid growth in population and industrial and agricultural outputs, as well as energy consumption and transportation infrastructures, during recent decades [25]. Earlier studies have reported that PAH contamination is often associated with the economic development and energy consumption in China [26,27]. In 2004, the emissions of PAHs in China (up to 114 Gg) accounted for an estimated 22% of total global PAH emissions [28]. Coal burning was responsible for 60% of PAH emissions in China [29]. The sedimentary records of organic contamination in lakes and marine areas have shown an increase in PAH quantities following the ~1850s, providing information with regard to regional fuel consumption and intensity of anthropogenic activities [14,30,31]. However, studies have mainly focused on developed regions, and limited information is available regarding the temporal trends of PAHs concentration within inland shallow lakes in the north of China. This leads to a significant lack of data about the impact of lifestyle and energy consumption on the concentration of PAHs.

Thus, it could be useful to evaluate the implications of the regional economic development and energy consumption on the temporal variation of PAH pollution to understand the factors related to the historical changes in PAH emissions [32,33]. The present study aims to (1) investigate the residual levels and temporal distribution of PAHs in sediment core from a typical inland shallow lake, Dongping Lake, located in the north of China, (2) speculate on the possible PAH sources in the undisturbed sediment profiles in combination with a detailed chronology study, and (3) reconstruct the historical trends of PAH contamination related to the economic development in the region (population size, gross domestic product, and energy consumption).

## 2. Materials and Methods

### 2.1. Study Area and Sampling

**Figure 1:**
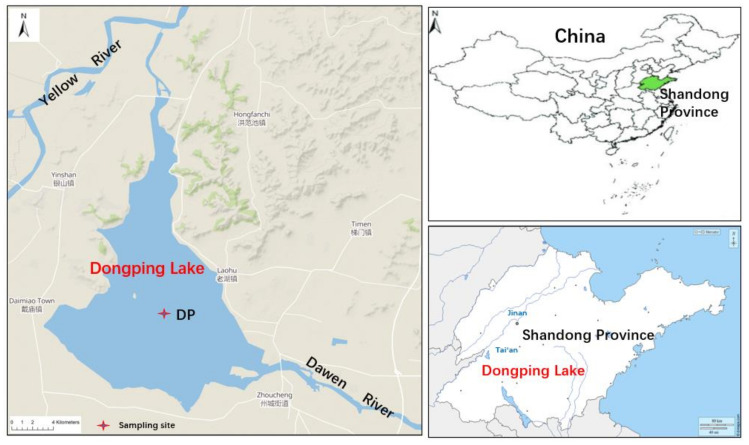
Map of the study area and location of the sampling site.

This study was carried out at Dongping Lake, a shallow freshwater lake with 627 km^2^ surface area and 4 × 10^9^ m^3^ storage volume, which is located in Tai’an city in the western part of Shandong province, China (35°30′–36°20′ N, 116°00′–116°30′ E) (Figure 1). Dongping Lake is the second largest freshwater source in Shandong province. Most of the lake area is no more than 3 m in depth. Dongping Lake is mainly affected by a warm and semi-humid continental monsoonal climate. The multi-annual mean temperature is 13.3 °C with an average annual precipitation of 640 mm [34]. Dongping Lake serves as an important flood control project in the lower reaches of the Yellow River and represents the last water reservoir along the Eastern Route of China's South-to-North Water Diversion Project [35]. The water in the lake flows north through the Xiaoqing River, eventually entering the Yellow River. Dawen River is the major inflow to Dongping Lake, whose recharge sources are mainly supplied by surface runoff and rainfall [36]. There has been increasing concern about water pollution by different types of contaminants in recent years, mainly due to the large inputs of industrial, agricultural, and urban sewage activities from the Dawen River. In recent years, the volume of sewage for the Dawen River reached 2.6 × 10^9^ tons, which has led to a severe degradation of the water quality of Dongping Lake [37].

The study was carried out in the autumn of 2016 in the Dongping Lake (Figure 1). Two parallel sediment cores with lengths of 60 cm were collected using a Beeker 04.23 core sampler (100 cm length × 57 mm ID; Eijkelkamp Co., Giesbeek, The Netherlands) at the still water area (DP: 35°57′23.1″ N, 116°11′35.6″ E) of Dongping Lake, where the water depth is approximately 3.0 m. The sediment core was sliced into 2 cm thick sections. Following this step, each section was placed in a precleaned aluminum foil, before being frozen, freeze-dried, ground into a fine homogenized powder, and finally stored at −20 °C until further treatment.

### 2.2. Total Organic Carbon Analysis and Sediment Core Dating

Total organic carbon (TOC) of sediment core samples was measured using a Perkin-Elmer PE 2400 Series II elemental analyzer. With the aim of dating the sediment core samples, the radioisotope activity concentrations (e.g.,^137^Cs, ^210^Pb, and ^226^Ra) in samples were determined using a well-type HPGe gamma detector (GCW3523, Canberra Inc., USA) at the Institute of Geology and Geophysics, Chinese Academy of Sciences. Before analyzing the radionuclides, each subsample was stored in sealed centrifuge tubes for 3 weeks, allowing radioactive equilibration [25]. The activities of ^137^Cs and ^210^Pb were respectively determined by the γ emissions at 662 keV and 46.5 keV, while the activity of ^226^Ra was determined by the γ emissions at 352 keV. Detection errors were within 5% for both ^137^Cs and ^210^Pb. ^210^Pbex was obtained by subtracting the ^226^Ra activity from the total ^210^Pb activity [38]. The ^210^Pb geochronology was calculated from a constant rate of supply (CRS) model [39] according to the following equation:

(1) $$t=\lambda^{-1}ln\left(A_{0}/A_{z}\right),\;$$

where A_0_ and A_z_ are the ^210^Pb_ex_ accumulation fluxes at the surface layer of the sediment core and depth *z*, respectively, while λ is the ^210^Pb_ex_ radioactive decay constant (0.03114 year^−1^). The profiles of ^137^Cs activity in samples were compared to scattering nuclides from nuclear testing, thermonuclear weapons testing in the middle 1960s, and nuclear accidents such as the Chernobyl nuclear site in 1986 [40]. Thus, the ^137^Cs activity was used as an independent chrono-marker to enhance the dating accuracy from ^210^Pb [24].

### 2.3. PAH Extraction and Analysis

The extraction of PAHs was performed according to a previously reported method [14]. Briefly, about 2.0 g (dw) of each sample was extracted with 250 mL of a dichloromethane–hexane mixture (1:1, *v*/*v*) for 24 h using a Soxhlet apparatus. A known mixture of surrogates (naphthalene-*d*8, acenaphthene-*d*10, phenanthrene-*d*10, and chrysene-*d*12) was added to each blank and sample before extraction. The extract passed through a glass column packed with 1:2 alumina–silica gel (*v*/*v*) containing 1 g of anhydrous sodium sulfate overlaying the silica gel. The eluents containing PAHs were collected by eluting 70 mL of hexane–dichloromethane (7:3, *v*/*v*) and were then concentrated to 1.0 mL. After adding a known quantity of an internal standard (hexamethylbenzene), the PAHs were analyzed by gas chromatography and mass spectrometry (GC/MS).

In the study, 16 PAHs, namely, naphthalene (Naph), acenaphthene (Aceph), acenaphthylene (Ace), fluorene (Fl), phenanthrene (Phen), anthracene (Ant), fluoranthene (Flu), pyrene (Pyr), benz[a]anthracene (BaA), chrysene (Chr), benzo[b]fluoranthene (BbF), benzo[k]fluoranthene (BkF), benzo[a]pyrene (BaP), dibenz[ah]anthracene (DBA), benzo[ghi]perylene (BgP), and indeno[1,2,3-cd]pyrene (InP), were detected. A Varian 4000 mass spectrometer (Varian Inc., Palo Alto, CA) coupled with a Varian CP-3800 gas chromatograph equipped with a Varian VF-5MS column (30 m × 0.25 mm × 0.25 μm) was employed to quantitatively determine PAHs. The column ramp temperature was programmed to rise from 80 °C (dwell time of 3 min) to 230 °C (dwell time of 2 min) with a rate of 15 °C/min, followed by a ramp to 290 °C (dwell time of 8 min) with a rate of 5 °C/min. The injection volume was 1 μL in splitless mode. All data were subject to strict quality control procedures. The spiked recoveries of 16 PAHs in samples were in the range of 75.3–107.4%. The method detection limits (MDLs) for each PAH ranged from 0.12 to 1.07 ng/g.

### 2.4. Positive Matrix Factorization (PMF) Model for Source Apportionment

The PMF model was used for source apportionment of sedimentary PAHs [33]. The model is based on an advanced multivariate factor analysis method that relies on weighted least squares calculation and was developed in 1994 [41]. The United States Environmental Protection Agency PMF user guide (version 5.0) explains the model in detail [42]. In theory, the PMF model can be described by Equation (2).

(2) $$X_{ij}=\overset{p}{\underset{j=1}{\sum }}g_{ik}f_{kj}+e_{ij},\;$$

where *X_ij_* is the concentration of the *i*-th species that was determined by the *j*-th sample, whereas, *g_ik_* is the *i*-th species concentration, which was detected in source *k*; *f_kj_* represents the contribution of the *k*-th source to the *j*-th sample, and *e_ij_* is the error for species *j* to sample *i* [43]. The objective function *Q(E)* of the PMF model is defined by Equation (3).

(3) $$Q\left(E\right)=\overset{n}{\underset{i=1}{\sum }}\overset{m}{\underset{j=1}{\sum }}[(X_{ij}-\overset{p}{\underset{k=1}{\sum }}g_{ik}k_{kj})/s_{ij}{]}^{2},$$

where *Q(E)* is the weighted sum of the squares for the difference in value between the original dataset and the PMF output [44], whereas *s_ij_* is the uncertainty in the *j*-th PAH to sample *i* [45].

### 2.5. Data Analysis

Origin Pro 8.0 was used to plot the experimental data. Statistical analyses were performed using SPSS 13.0 (SPSS Inc., Microsoft Co., USA). The correlation coefficients between the measured parameters were calculated through a two-tailed test and the Pearson correlation coefficient.

## 3. Results and Discussion

### 3.1. Sediment Chronology

**Figure 2:**
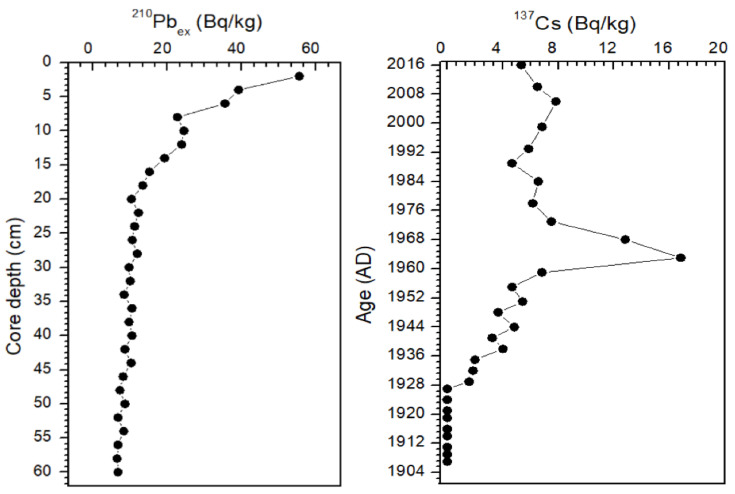
Age–depth profiles of excess ^210^Pb (^210^Pb_ex_) and ^137^Cs in the sediment core.

Figure 2 shows the vertical distribution of excess ^210^Pb (^210^Pb_ex_) and ^137^Cs for the sediment core collected from Dongping Lake. The ^137^Cs activity was low (<17 Bq/kg) throughout the sediment core; however, a typical peak of ^137^Cs activity (16.8 Bq/kg) could be identified at 22 cm depth. This corresponded well with the ^137^Cs atmospheric fallout peak from the nuclear bomb testing in 1963 [46]. The ^210^Pb_ex_ activity showed a continuous increase from 6.7 Bq/kg (at the core bottom) to 55.5 Bq/kg (at the core top). The ^210^Pb_ex_ activity profile showed a definite exponential decay together with increasing depth (*R*^2^ = 0.934); thus, a CRS dating model was applied to date the sediment core to determine the chronologies of ^210^Pb [39]. The ^210^Pb_ex_ CRS model provided an age of 1963 at the depth of 22 cm, which was verified by the age provided by the independent ^137^Cs dating peak. Mover, the enrichment factor of Pb significantly increased after 1960 due to gasoline-related Pb emission [47], which was consistent with the historical trend of ^210^Pb_ex_ activity in sediment core. Thus, the chronology based on the ^210^Pb_ex_ CRS model was considered reliable. According to this age–depth model, the estimated average sedimentation rate was approximately 0.56 cm/year at the site, meaning that the 60 cm core covered ~110 years of sedimentary history between 1907 and 2016. 

It is noted that ^210^Pb_ex_ activity was higher than found in earlier studies from the adjacent areas, for instance, those from Baiyangdian Lake in Hebei Province [48] and Lake Gonghai in Shanxi Province [23]. This is because the ^210^Pb_ex_ activity in sediment cores is positively correlated with the atmospheric deposition flux [23]. The average sediment accumulation rate in the sediment core from Lake Gonghai (0.17 g/cm^2^/year) [23] was lower than that performed at the Lake Chaohu (0.23 g/cm^2^/year) [49] and the Dongping Lake (0.22 g/cm^2^/year) [50]. In addition, atmospheric deposition of ^210^Pb is also influenced by local precipitation. The climate between the two sites is also quite different, with an annual average precipitation of 456 mm at Lake Gonghai and 680 mm at Dongping Lake, which could lead to large variance of the ^210^Pb_ex_ activity in the sediment cores.

### 3.2. Temporal Variation of PAH Concentration and Composition

**Figure 3:**
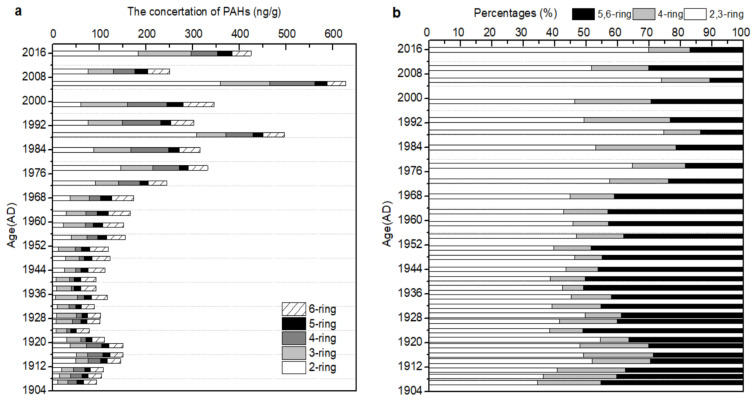
The historical variations of the concentrations (**a**) and percentages (**b**) of polycyclic aromatic hydrocarbons (PAHs) with different rings (2–6 rings) in the sediment core of Dongping Lake. Two-ring PAHs include Naph; three-ring PAHs include Aceph, Ace, Fl, Phen, and Ant; four-ring PAHs include Flu, Pyr, BaA, and Chr; five-ring PAHs include BbF, BkF, BaP, and DBA; six-ring PAHs include InP and BgP.

The concentration of PAHs at different dates of the lake’s sediment core is illustrated in Table S1 and Figure 3a. All 16 priority PAHs were detected in the sediment core back to the 1970s, yet only nine individual PAHs were detected before the 1970s in the totality of the core samples. Increased industrial and agricultural activity after the 1970s may have increased the input of PAHs such as Ace, Fl, and DBA [14]. An overall increasing trend of total PAH concentration (ΣPAHs) was observed from 1907 to 2016, and the ΣPAH concentration ranged from 77.6 to 628.0 ng/g. Three temporal trends for the concentration of ΣPAHs in the sediment core were characterized. In the first stage from 1907 to the 1950s, the concentration of ΣPAHs spread over a relatively narrow range (77.6–122.1 ng/g) showing a similar constant trend before the mid-20th century. In the second stage from the 1950s to 2006, the concentrations of ΣPAHs increased sharply, reaching around 628.0 ng/g in ca. 2006. This increase can be attributed to rapid industrialization and urbanization following the establishment of the People’s Republic of China in 1949, as well as the implementation of the Reform and Open Policy in 1978 [26]. Moreover, the construction of the Dongping Industrial Park in 2002–2005 and the consequently increasing development activities around the lake not only further deteriorated the water quality of Dongping Lake [51], but also increased the input of PAHs. In the third stage from 2006 to 2016, the concentrations of ΣPAHs (average value of 337.6 ng/g) decreased compared to the period of 2000–2006. The implementation of pollution control measures, which were carried out to guarantee the water quality and safety of the South-to-North Water Diversion in the catchment since the 2005, may have already reduced the emissions of PAHs and deposition in the lake sediment [36]. Compared with other lakes subject to more frequent industrial and human activities in China, Dongping Lake is a lake mainly developed for agriculture and tourism, and the peak (455.4 ng/g) PAHs in this location were lower than observed in Dianchi Lake (4560.8 ng/g) [52], Chaohu Lake (2500 ng/g) [32], and Taihu Lake (1600 ng/g) [30]. In addition, the peak period (in the 1990s–2000s) of PAHs in Dongping Lake appeared later than that in the aforementioned lakes (in the 1980s–1990s). The different temporal trends of the ΣPAH concentration in the sediment core of lakes in China indicated different histories of industrialization and development intensity in different catchments.

Different types of PAHs were grouped according to their number of aromatic rings, and the concentration and percentage of PAHs ranging from 2–6 rings were calculated (Figure 3). The concentration of two- and four-ring PAHs slowly increased from the 1900s to the 1920s, and then decreased and maintained a steady value between the 1920s and the 1950s, finally showing a significantly increasing trend after the 1950s. Concentrations of three-, five-, and six-ring PAHs increased from the 1900s to recent years, with the three-ring PAH concentration increasing even more. The 2,3-ring PAHs exhibited the highest ratios to total PAHs compared to other multiring PAHs, considering the period from the 1970s to the recent years, with an average of 60.1% over the last four decades. The two total PAHs peaks in 1989 and 2006 had the highest 2,3-ring PAH contribution to the total PAH amount (>74%), suggesting that the more frequent and high-intensity occurrence of petrogenic discharge and low–moderate temperature combustions (e.g., incomplete grass and wood burning) allegedly occurred in these periods [32,51]. Compared with the early and late 2000s, the water level of Dongping Lake in 2006 was at a low level, which reduced the water environment capacity and further deteriorated the water quality [53]. In addition, according to the statistical data of Tai’an city, the amount of biomass combustion in 2006 was the highest during the 2000s, which was twice that in 2010 [54], which resulted in more 2,3-ring PAHs entering the lake through atmospheric emissions [32]. These factors resulted in the highest concentration of PAHs and a high proportion of 2,3-ring PAHs in 2006 (Figure 3). This study further confirmed that the deposited PAHs found in the sediment core of the lake are strictly derived from the transportation and precipitation of atmospheric aerosols [55] and direct emissions from petrogenic sources [23], as observed in some lakes of rural areas in Thailand [24,56], as well as in Baiyangdian Lake [14] and Yangzonghai Lake [25] from China.

Furthermore, the relationship between PAH concentration and TOC content in sediment cores was investigated using Pearson’s coefficient rank correlation. A significant positive correlation was found between PAH and TOC concentrations at the 0.05 level of significance (*r* > 0.7, *p* < 0.01, *n* = 30), with correlation coefficients of 0.776, 0.905, and 0.859 for 2,3-ring PAHs, four-ring PAHs, and 5,6-ring PAHs, respectively. This indicates that PAH pollution in sediment core was primarily controlled by TOC. Several previous studies have also demonstrated that the TOC in the sediment is an important indicator for determining the fate, sorption dynamics, and sequestration mechanisms of PAHs [19,57,58]. The correlation between TOC and four-ring or 5,6-ring PAH concentration was better compared to that between TOC and 2,3-ring PAH concentration. This may be attributed to the strong hydrophobicity and degradation resistance of four-ring or 5,6-ring PAHs [23,59].

### 3.3. Sources Analysis

**Figure 4:**
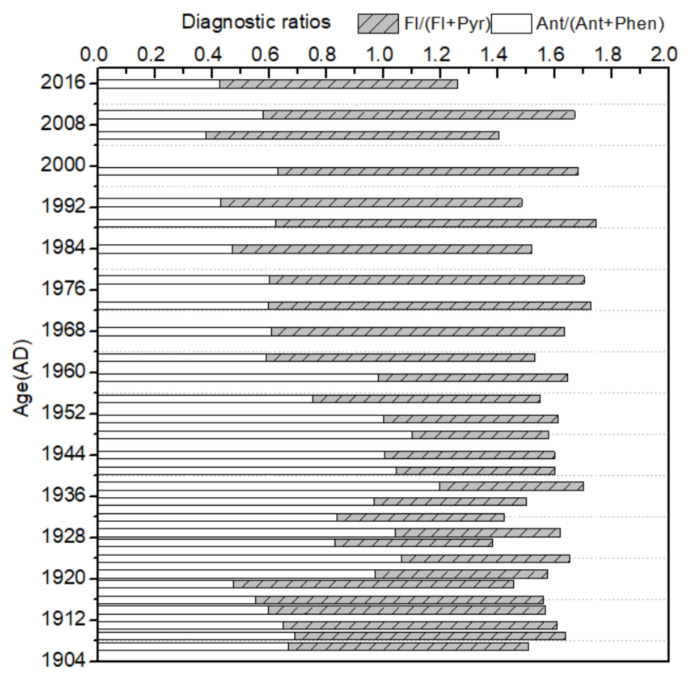
Diagnostic ratios of polycyclic aromatic hydrocarbon (PAH) ratios in the sediment core of Dongping Lake.

Molecular diagnostic ratios of specific PAHs were applied to identify a more specific origin of PAHs found in the environment [32]. Many former studies used this methodology to confirm the possible emission sources of PAHs [14,60,61]. PAH molecular ratios for Ant/(Ant + Phen) and Fl/(Fl + Pyr) were applied to calculate the possible sources of PAHs. The results for this study are shown in Figure 4. Ant/(Ant + Phen) ratios lower than 0.1 suggest that the PAHs principally originated from petrogenic source. Ratios higher than 0.1 are considered representative of pyrogenic sources including biomass, coal, and petroleum combustion [25,60]. Fl/(Fl + Pyr) ratios less than 0.4 are often considered to be typical of petroleum contamination. Ratios greater than 0.5 imply that PAH compounds were primarily generated from pyrogenic sources, especially grass, wood, and coal burning, while values of Fl/(Fl + Pyr) that fall between 0.4 and 0.5 point to liquid fossil-fuel combustion [58,62]. For the Ant/(Ant + Phen) ratios, all values were >0.1 in the sediment core, which indicates a combustion source. The Fl/(Fl + Pyr) ratios showed the two types of PAHs sources. The values exceeded the threshold of 0.5 from the 1907s to the 1920s and from the 1950s to the recent years, which suggests a strong pyrogenic signal from grass, wood, and coal combustion. For the period ranging from 1907 to the 1920s, the pyrogenic source was mainly from the combustion of grass and wood related to natural or anthropogenic wildfire. For the period from the 1950s to recent years, the pyrogenic source was mainly related to the combustion of biomass and coal due to the increasing industrial activities since the 1950s. Further studies have shown that the contribution of coal and biomass combustion is about 44% and 24% to the PAHs found in the Shandong province, respectively [27]. Thus, the residential indoor wood and crop burning and coal combustion may be the two major emission sources of PAHs in the region. The Fl/(Fl + Pyr) ratios ranged from 0.38 to 0.49, corresponding to the period of time of the 1920s to the 1950s, reflecting PAHs from liquid fossil-fuel combustion. This might show the effect of the Chinese Liberation War (1946–1949), World War II (1937–1945), and the Chinese Civil Revolutionary War (1927–1937) on the temporal distribution of PAHs [14].

### 3.4. The Impact of Economic Parameters on the Change in PAH Concentrations and Sources

**Figure 5:**
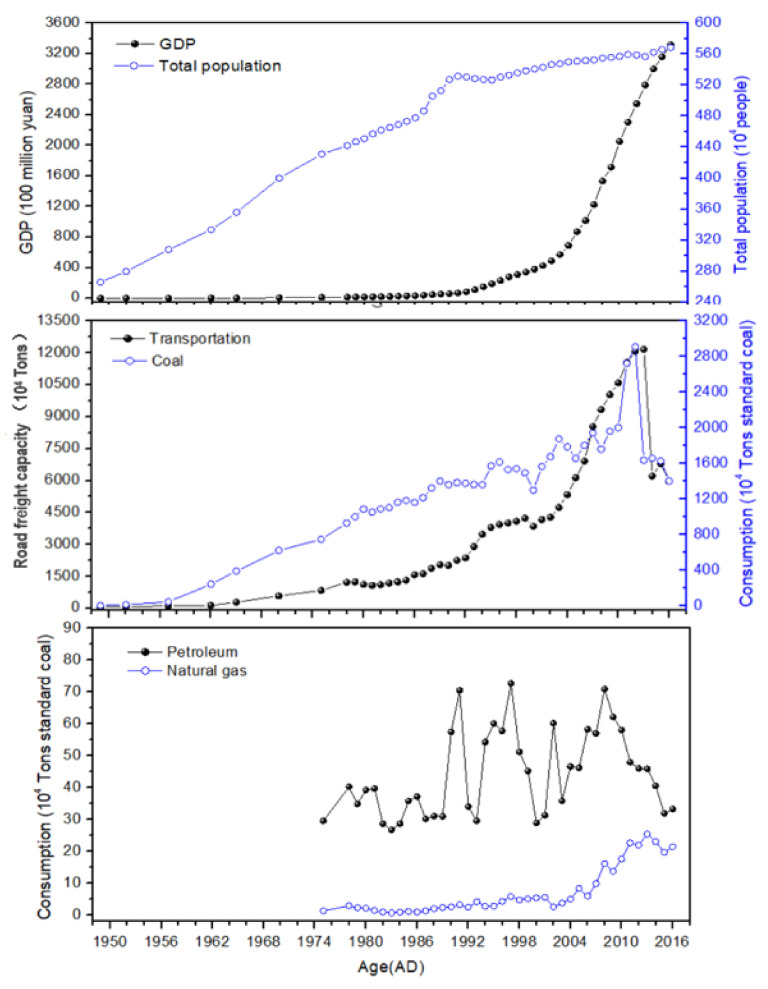
Statistical data on the socioeconomic development and energy consumption in Tai’an city.

Historical changes in the concentrations of PAHs in this study followed the general temporal trends reported from the socioeconomic development data in Tai’an city in Shandong province. Due to the limitation of historical statistics, the only used data were gross domestic product (GDP), total population, rural population, road freight capacity, and coal consumption data from 1949 to 2016; similarly, petroleum consumption and natural gas consumption data from 1975 to 2016 were used (Figure 5) [54]. According to Figure 3a, the concentration of ΣPAHs increased sharply from the 1950s to 2006, and then decreased from 2006 to recent years. Since the founding of the People’s Republic of China in 1949, an increase in population from 2.7 × 10^6^ to 5.7 × 10^6^ people has been observed, along with increases in GDP from 1.0 × 10^2^ to 3.3 × 10^5^ million yuan, coal consumption from 6.4 × 10^4^ to 2.9 × 10^7^ tons standard coal, and road freight capacity from 6.6 × 10^5^ to 1.2 × 10^8^ Tons in Tai’an city; this growth has contributed to an increase and accumulation of PAHs in the catchments, with a good correlation at the 0.05 level of significance (*r* > 0.75, *p* < 0.01, *n* = 15). The intensification of the industrial activities around lake areas such as the Dongping Industrial Park development since 2002 [51], combined with the impact of pollutant transmission phenomena in the area, allowed the concentration of PAHs to reach a peak in 2006. Later, as the Chinese government carried out environmental protection and implemented energy conservation and emissions reduction [14], together with the development of the South-to-North Water Diversion Project in the region [36], the emissions of PAHs were effectively reduced, leading to a decline in the accumulation of PAHs in the lake. For example, the road freight capacity gradually decreased from 1.2 × 10^8^ to 6.0 × 10^7^ tons, with the coal consumption decreasing from 2.9 × 10^7^ to 1.4 × 10^7^ tons of standard coal and the petroleum consumption decreasing from 6.2 × 10^5^ to 3.2 × 10^5^ tons of standard coal (Figure 5).

The relationship between the vertical distribution of the concentration of different-ring PAHs and energy consumption (Figure 5) in the period of 1975–2016 was further analyzed. With regard to the consumption of coal from 1975 to 2016, the value gradually increased, reaching a peak in 2012 and then gradually faded. On the other hand, petroleum consumption was relatively stable at a value (3.3 × 10^5^ tons standard coal) before the 1990s, and then fluctuated between this latter value and 3.0–7.1 × 10^5^ tons standard coal. The consumption of natural gas augmented slowly from 0.7 to 8.4 × 10^4^ tons standard coal between 1975 and 2006, and then increased rapidly to 2.5 × 10^5^ tons standard coal. The results shown in Figure 3 and Figure 5 indicate that the increase in concentration for the 2,3-ring and four-ring PAHs was consistent with the surge of coal consumption, thus suggesting that the household energy usage structure was a major factor impacting the concentration of PAHs [33]. Although coal consumption has declined since 2010, it is still the main structural energy source in the region, accounting for 96.7% of the overall energy structure [54]. The correlation coefficients between the concentration of 2,3-ring and four-ring PAHs and coal consumption reached values of 0.75 and 0.91, respectively, between 1975 and 2016. Coal consumption emits higher levels of 2,3 ring and four-ring PAHs compared to the burning of petroleum products or natural gas [33,63]. A comparison of the concentrations of 5,6-ring PAHs with petroleum consumption may reflect a relationship between PAH pollution and vehicles exhaust emissions [33]. The correlation coefficient calculated between the concentration of 5,6-ring PAHs and the petroleum consumption was 0.85 between 1975 and 2016. The positive relationship between 5,6-ring PAHs and the traffic volume was observed to be 0.89 between 1975 and 2016, confirming once again this relationship. Moreover, the concentrations of different-ring PAHs had a good correlation with the natural gas consumption at the 0.05 level of significance (*r* > 0.61, *p* < 0.01, *n* = 15), suggesting that natural gas is gradually becoming a new source of contribution to PAH pollution.

**Figure 6:**
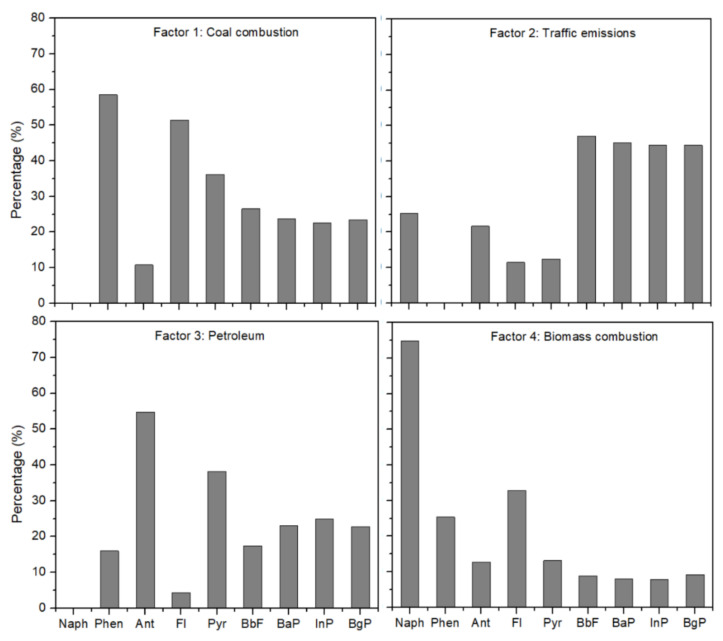
Four main source components of sedimentary polycyclic aromatic hydrocarbons (PAHs) obtained using a positive matrix factorization (PMF) model for Dongping Lake.

The main source components were classified according to the data of Naph, Phen, Ant, Fl, Pyr, BbF, BaP, InP, and BgP having the largest contribution for the period from the 1970s to 2016; the PMF source file is shown in Figure 6. There are four factors extracted by PMF model; the percentage contributions to PAH sources were 51.4% by Factor 1, 11.5% by Factor 2, 4.3% by Factor 3, and 32.8% by Factor 4. Factor 1 showed high levels of Phen, Fl, and Pyr, suggesting that this factor might be coal combustion [33,63]. Moreover, a high correlation coefficient (*R*^2^ = 0.81) between Factor 1 contributions and the total consumption of coal from the 1970s to 2016 was observed. Factor 2 had high levels of BbF, BaP, InP, and BgP (i.e., high-molecular-weight PAHs), indicating this factor as a possible marker of gasoline and diesel emissions [64,65]. The correlation coefficient of Factor 2 contributions and road freight capacity was 0.55, which further confirmed Factor 2 as likely related to traffic emissions. Factor 3 correlated strongly with Ant and Pyr, which is associated with refined petroleum combustion or crude oil leakage [14]. The correlation coefficient between Factor 3 contributions and the total consumption of petroleum was 0.53, indicating that this factor might represent petrogenic sources. Factor 4 showed a high loading of Naph and Fl and was identified as an important indicator of biomass combustion [4,63]. Furthermore, the correlation coefficient between Factor 4 contribution and the rural population reached 0.66, suggesting that the factor might represent the suburban lifestyle of biomass burning for cooking and heating [33]. According to the PMF results, four sources were successfully identified: (1) coal combustion sources (51.4% of total factor contributions), (2) traffic emissions (11.5% of total), (3) petrogenic sources (4.3% of total), and (4) biomass combustion and contribution (32.8% of total). Hence, coal combustion was recognized as the dominant source of PAHs in Dongping Lake in the last four decades.

## 4. Conclusions

The historical variation of PAH pollution in the sediment core of Dongping Lake was investigated in this study. The concentration of the ΣPAH_16_ fluctuated from 77.6 to 628.0 ng/g (with a mean value of 198.3 ng/g), which was significantly lower compared to the lakes located in the areas with frequent industrial and human activities. The ΣPAH16 was mainly composed of 2,3-ring PAHs (48.7%), followed by 5,6-ring PAH (35.4%) and four-ring PAHs (15.9%). The concentration of 2,3-ring, four-ring, and 5,6-ring PAHs varied from 32.3 to 464.5 ng/g, 6.1 to 96.8 ng/g, and 39.6 to 101.8 ng/g, respectively. Since the 1970s, the increase in population and the construction of industrial parks have promoted the accumulation of PAHs in the area. In addition, the main energy structure significantly affects the input and composition of PAHs. The molecular diagnostic ratios of specific PAHs demonstrated that pyrogenic sources were the main PAH sources in the sediment core from Dongping Lake, with coal combustion (51.4% of total sources contributions) and biomass combustion (32.8% of total) being the dominant sources of PAHs since the 1970s, according to analysis of the PMF model. The results indicate that an appropriate adjustment of regional energy structure and encouragement of clean energy use can help reduce the impact of PAHs on lakes, improve air quality, and reduce carbon emissions.

## Data Availability

Not applicable.

## Supplementary Materials

The following are available online: Table S1. Concentration of PAHs and total organic carbon (TOC) in sediment core from the Dongping Lake.
