# Supplementary material for: A 110 Year Sediment Record of Polycyclic Aromatic Hydrocarbons Related to Economic Development and Energy Consumption in Dongping Lake, North China
Source: Molecules. 2021 Nov 11;26(22):6828. doi: 10.3390/molecules26226828 (PMC8622884; doi:10.3390/molecules26226828)
Supplement: Supplementary file 1 [file molecules-26-06828-s001.zip › molecules-1441512-supplementary.pdf]

molecules

MDPI

Supplementary Materials

A 110-Year Sediment Record of Polycyclic Aromatic Hydrocarbons Related to the Economic Development and Energy Consumption in Dongping Lake, North China

Wei Guo [1,*], Junhui Yue [1], Qian Zhao [1], Jun Li [1], Xiangyi Yu [2] and Yan Mao [2,*]

[1] Key Laboratory of Beijing for Water Quality Science and Water Environment Recovery Engineering, Beijing University of Technology, Beijing 100124, China
[2] Solid Waste and Chemicals Management Center of MEE, Beijing, 100029, China
* Correspondence: gwfybj@bjut.edu.cn (W. Guo); maoyan@meescc.cn (Y. Mao)

Table S1. Concentration of PAHs and total organic carbon (TOC) in sediment core from the Dongping Lake.

Year	Naph	Acep h	Ace	Flu	Phen	Ant	Fl	Pyr	BaA	Chr	BbF	BkF	BaP	InP	DBA	BgP	ΣPA Hs	TOC
	ng/g	ng/g	ng/g	ng/g	ng/g	ng/g	ng/g	ng/g	ng/g	ng/g	ng/g	ng/g	ng/g	ng/g	ng/g	ng/g	ng/g	%
2016	182.1	9.0	47.6	4.2	35.1	18.1	35.1	17.3	2.9	0.9	11.1	5.9	11.5	25.5	2.8	15.3	424.5	1.47
2010	75.8	3.3	4.3	2.2	23.7	20.3	37.5	5.4	1.6	1.1	10.3	3.5	12.7	29.3	1.6	18.1	250.7	1.51
2006	359.1	17.2	5.5	2.3	56.3	24.2	76.9	16.6	1.8	1.5	9.4	3.1	11.3	25.0	2.4	15.4	628.0	1.89
1999	60.2	5.9	6.6	4.1	46.7	47.3	70.6	13.3	1.8	1.1	14.1	3.6	18.0	41.5	1.2	24.6	360.6	2.14
1993	76.1	8.4	4.8	2.6	42.2	22.0	69.9	12.9	1.6	1.4	7.7	2.9	12.9	32.7	2.5	17.4	318.0	1.61
1989	308.2	8.0	4.1	2.9	27.3	27.1	52.0	6.0	2.5	2.1	9.1	3.8	12.5	29.3	3.2	16.7	514.8	1.34
1984	88.1	10.0	7.8	4.1	43.2	26.1	67.5	13.0	2.1	0.9	9.6	2.1	12.7	29.7	2.1	15.8	334.8	1.27
1978	144.9	7.6	6.6	3.2	32.2	29.6	49.8	6.6	1.7	1.2	8.3	1.8	11.0	27.4	1.5	14.4	347.8	0.96
1973	91.0	5.3	3.3	2.0	23.0	20.9	41.7	4.4	1.3	0.8	7.4	1.6	10.9	26.1	1.2	13.7	254.6	0.88
1968	36.6	nd	nd	nd	21.3	20.0	20.1	4.3	nd	nd	10.1	nd	13.4	31.0	nd	16.6	173.6	0.72
1963	28.9	nd	nd	nd	22.3	19.8	17.6	5.8	nd	nd	10.5	nd	13.6	30.5	nd	17.3	166.2	0.77
1959	22.4	nd	nd	nd	10.1	36.8	9.1	8.0	nd	nd	8.0	nd	12.0	29.7	nd	15.5	151.7	0.42
1955	39.7	nd	nd	nd	13.1	19.8	14.8	8.4	nd	nd	8.4	nd	11.1	26.2	nd	13.6	155.0	0.59
1951	11.2	nd	nd	nd	7.2	28.9	6.9	7.1	nd	nd	7.1	nd	10.6	26.6	nd	13.7	119.4	0.43
1948	26.8	nd	nd	nd	3.6	26.1	4.1	6.6	nd	nd	6.6	nd	10.4	25.0	nd	13.0	122.1	0.55
1944	24.5	nd	nd	nd	4.8	19.3	5.5	6.0	nd	nd	6.0	nd	9.7	23.8	nd	12.2	111.8	0.48
1941	6.4	nd	nd	nd	4.8	24.3	4.5	5.6	nd	nd	5.6	nd	8.4	21.2	nd	11.0	91.9	0.52
1938	8.4	nd	nd	nd	1.3	29.7	3.3	5.0	nd	nd	5.0	nd	8.7	22.2	nd	11.4	95.0	0.26
1935	5.6	nd	nd	nd	10.7	36.2	4.5	6.1	nd	nd	6.1	nd	9.2	22.2	nd	11.4	112.1	0.35
1932	8.9	nd	nd	nd	8.5	17.2	4.5	5.1	nd	nd	5.1	nd	7.5	18.2	nd	9.4	84.2	0.38
1929	8.0	nd	nd	nd	7.1	35.5	4.1	4.8	nd	nd	4.8	nd	7.5	18.2	nd	9.3	99.3	0.35
1927	6.4	nd	nd	nd	12.0	23.5	4.2	5.4	nd	nd	5.4	nd	7.7	18.3	nd	9.3	92.1	0.32
1924	7.1	nd	nd	nd	3.4	19.2	3.9	4.4	nd	nd	4.4	nd	7.4	18.4	nd	9.4	77.6	0.21
1921	30.0	nd	nd	nd	6.8	23.6	4.8	5.2	nd	nd	5.2	nd	7.8	18.2	nd	9.4	111.0	0.25
1919	37.4	nd	nd	nd	21.5	13.2	25.6	7.1	nd	nd	7.1	nd	8.6	19.2	nd	10.4	150.0	0.28
1916	50.2	nd	nd	nd	13.2	10.4	27.0	6.5	nd	nd	6.5	nd	8.3	18.9	nd	9.6	150.6	0.37
1914	48.6	nd	nd	nd	13.9	12.7	21.0	6.0	nd	nd	6.0	nd	8.2	18.8	nd	10.0	145.3	0.32
1911	18.7	nd	nd	nd	12.4	13.3	18.0	5.4	nd	nd	5.4	nd	7.8	18.3	nd	9.4	108.5	0.48
1909	13.5	nd	nd	nd	10.8	13.3	18.4	5.9	nd	nd	5.9	nd	7.5	18.7	nd	9.9	103.9	0.31
1907	10.9	nd	nd	nd	10.0	11.5	12.7	6.2	nd	nd	6.2	nd	8.2	18.4	nd	9.6	93.6	0.27

Naph, naphthalene; Aceph, acenaphthene; Ace, acenaphthylene; Flu, fluorene; Phen, phenanthrene; Ant, anthracene; Fl, fluoranthene; Pyr, pyrene; BaA, benz[a]anthracene; Chr, chrysene; BbF, benzo[b]fluoranthene; BkF, benzo[k]fluoranthene; BaP, benzo[a]pyrene; DBA, dibenz[ah]anthracene; BgP, benzo[ghi]perylene; InP, indeno[1,2,3-cd]pyrene; nd, not detected.
